# 4β-Hydroxycholesterol is a prolipogenic factor that promotes SREBP1c expression and activity through the liver X receptor

**DOI:** 10.1016/j.jlr.2021.100051

**Published:** 2021-02-23

**Authors:** Ofer Moldavski, Peter-James H. Zushin, Charles A. Berdan, Robert J. Van Eijkeren, Xuntian Jiang, Mingxing Qian, Daniel S. Ory, Douglas F. Covey, Daniel K. Nomura, Andreas Stahl, Ethan J. Weiss, Roberto Zoncu

**Affiliations:** 1 Department of Molecular and Cell Biology, University of California at Berkeley, Berkeley, CA, USA; 2 The Paul F. Glenn Center for Aging Research, University of California, Berkeley, Berkeley, CA, USA; 3 Cardiovascular Research Institute, UCSF, San Francisco, CA, USA; 4 Department of Nutritional Sciences and Toxicology, University of California at Berkeley, Berkeley, CA, USA; 5 Diabetic Cardiovascular Disease Center, Washington University School of Medicine, St Louis, MO, USA; 6 Department of Developmental Biology, Washington University School of Medicine, St Louis, MO, USA

**Keywords:** oxysterol, SREBP1c, liver-X-Receptor, de-novo-lipogenesis, lipid droplets, insulin, 4β-HC, 4β-hydroxycholesterol, DNL, de novo lipogenesis, DPBS, dulbecco’s phosphate buffered saline, *ent*-4HC, enantiomer of 4β-HC, EtOAc, ethyl acetate, HC, hydroxycholesterol, LD, lipid droplet, LDS, lipid-depleted serum, mTOR, mechanistic Target of Rapamycin, NAFLD, nonalcoholic fatty liver disease, PI3K, phosphatidylinositol 3-kinase, THF, tetrahydrofuran

## Abstract

Oxysterols are oxidized derivatives of cholesterol that play regulatory roles in lipid biosynthesis and homeostasis. How oxysterol signaling coordinates different lipid classes such as sterols and triglycerides remains incompletely understood. Here, we show that 4β-hydroxycholesterol (HC) (4β-HC), a liver and serum abundant oxysterol of poorly defined functions, is a potent and selective inducer of the master lipogenic transcription factor, SREBP1c, but not the related steroidogenic transcription factor SREBP2. By correlating tracing of lipid synthesis with lipogenic gene expression profiling, we found that 4β-HC acts as a putative agonist for the liver X receptor (LXR), a sterol sensor and transcriptional regulator previously linked to SREBP1c activation. Unique among the oxysterol agonists of the LXR, 4β-HC induced expression of the lipogenic program downstream of SREBP1c and triggered de novo lipogenesis both in primary hepatocytes and in the mouse liver. In addition, 4β-HC acted in parallel to insulin-PI3K–dependent signaling to stimulate triglyceride synthesis and lipid-droplet accumulation. Thus, 4β-HC is an endogenous regulator of de novo lipogenesis through the LXR-SREBP1c axis.

All cells must achieve and maintain a balanced composition of their internal membranes to grow, proliferate, or adapt to sudden changes in external conditions and nutrient availability (1). Dedicated biosynthetic pathways mediate the synthesis of fatty acids, sterols, phospholipids, and sphingolipids, but how these pathways communicate with each other to coordinate their respective activities and respond to changing metabolic needs is poorly understood (2, 3).

The liver X receptor (LXR) α and β are transcription factors belonging to the nuclear receptor superfamily that play key roles in maintaining lipid homeostasis in multiple cells and organs (4, 5, 6, 7). The LXRα and LXRβ dimerize with the retinoid X receptor (RXR) and activate target genes that mediate cholesterol efflux from cells, including ABC-family transporters, as well as genes that mediate conversion of cholesterol into bile acids in the liver to facilitate cholesterol elimination from the body, such as cytochrome p450 7a-hydroxylase (8, 9, 10). Accordingly, mice lacking the *LXRα* exhibit impaired bile acid metabolism and defective cholesterol elimination (9), along with enhanced inflammation and formation of atherosclerotic plaques (11). Conversely, synthetic LXRα agonists have shown promise in reducing atherosclerosis and preventing cardiovascular disease in animal models (12, 13, 14).

Another key mediator of lipid homeostasis is the helix-loop-helix-leucine zipper transcription factor, SREBP1c. SREBP1c is a master regulator of biosynthesis of fatty acids and triglycerides [collectively referred to as de novo lipogenesis (DNL)] that is subject to tight transcriptional and posttranslational regulation. Along with its paralogue, the master steroidogenic transcription factor SREBP2, SREBP1c resides at the endoplasmic reticulum (ER) membrane, to which it is anchored via a single transmembrane helix. When cholesterol concentration in the ER membrane is low, SREBP1c and SREBP2 are transported to the Golgi apparatus via interaction with SREBP cleavage-activating protein, a cholesterol-sensing chaperone that favors their loading into COPII vesicles. At the Golgi membrane, resident proteases cleave the DNA-binding portion of SREBP1c and SREBP2 from the transmembrane portion, enabling their translocation to the nucleus and activation of downstream programs for DNL and de novo steroidogenesis, respectively.

In addition to their homeostatic regulation by cholesterol levels, the SREBPs lie downstream of metabolic hormone signaling. For example, in the liver, both the expression and proteolytic activation of SREBP1c are stimulated by the insulin-phosphatidylinositol 3-kinase (PI3K)-mechanistic Target of Rapamycin (mTOR) pathway, as part of a mechanism that converts excess of glucose into lipids, which are required for energy storage (15, 16, 17). However, the range of regulatory inputs to SREBP1c and their respective interplay remain to be fully elucidated.

The LXRα and LXRβ were shown to directly bind to the promoter of the *SREBP1c* gene and trigger activation of its downstream lipogenic genes (6). Accordingly, synthetic LXR ligands strongly promote DNL and increased plasma triglyceride levels (13, 18, 19), providing evidence for cross-talk between LXR- and SREBP1c-dependent programs.

Although the physiological significance of LXR-dependent regulation of DNL through SREBP1c remains unclear, this cross-talk has important clinical implications. In particular, LXR-dependent upregulation of SREBP1c potentially limits the usefulness of LXR agonists to improve cholesterol metabolism, as the resulting induction of lipogenic programs could lead to undesirable effects, such as nonalcoholic fatty liver disease (NAFLD), a condition that has risen to epidemic proportions in recent years (20). Thus, understanding how LXR-dependent activation of *SREBP1c* occurs and its functional interaction with other pathways controlling lipid homeostasis such as PI3K-mTOR signaling are key open questions.

Oxysterols are a family of metabolites that originate from an oxygenation reaction of cholesterol. Some oxysterols are signaling molecules involved in a wide range of physiological processes controlling cholesterol, glucose, and lipid metabolisms (21). Levels of oxysterols are known to change in pathological situations such as obesity, atherosclerosis, and Alzheimer's disease (22, 23). A subset of oxysterols function as endogenous LXR ligands and were shown to activate LXRα-dependent gene expression in vitro, including those bearing hydroxyl groups in positions 4, 7, 20, 22, 24, 25, and 27 on the cholesterol backbone (4, 24, 25). Interestingly, although these oxysterols are considered bona fide LXR activators, none is known to activate *SREBP1c* and its downstream lipogenic programs, whereas several oxysterols have been shown to promote LXR-dependent cholesterol efflux. In contrast, synthetic LXR ligands including T0901317 and GW3965 can induce both cholesterol efflux and SREBP1c-dependent DNL (18, 19). This leads to the question of whether DNL is a physiologically relevant LXR-dependent response, and if so, the identity of the endogenous ligand that triggers LXR-dependent *SREBP1c* expression.

Here we identify 4β-hydroxycholesterol (HC) (4β-HC) as an LXR activator that selectively triggers *SREBP1c* activation and de novo fatty acid and triglyceride synthesis. 4β-HC promoted the expression and proteolytic processing of SREBP1c but not of the related steroidogenic factor SREBP2, thus triggering de novo synthesis of fatty acids but not cholesterol. In primary mouse hepatocytes, 4β-HC additively enhance insulin action in promoting *SREBP1c* expression and activation, leading to increased triglyceride synthesis and storage. Thus, 4β-HC may be a novel lipogenic factor that can shift lipid homeostasis toward triglyceride accumulation via regulation on *SREBP1c*.

## Results

### 4β-HC is a unique oxysterol that drives SREBP1c gene expression

**Fig. 1:**
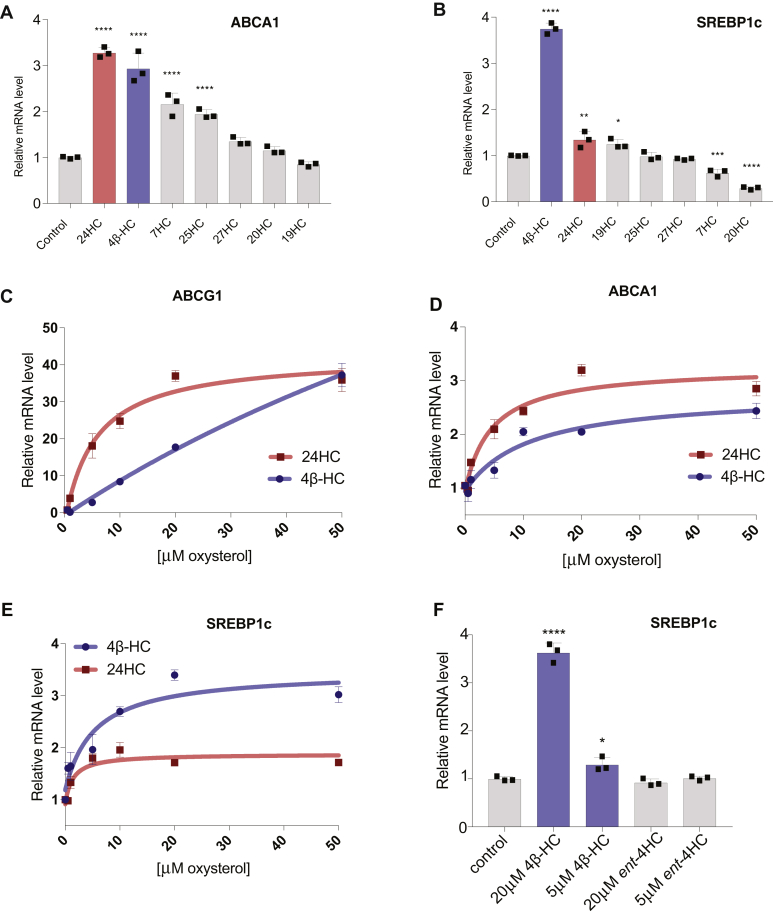
4β-HC is a unique LXR ligand that drives *SREBP1c* expression. A: Oxysterol screen for LXR target gene expression. Huh7 cells were treated with indicated oxysterols (20 μM) in 24-h time course. ABCA1 mRNA levels or (B) SREBP1c mRNA level were measured by RT-PCR (N = 3). C: Dose-response curves of 4β-HC and 24-HC in Huh7 cells were treated for 24 h. mRNA levels of ABCA1, (D) ABCG1, and (E) SREBP1c were measured by RT-PCR. Line plotted by nonlinear fit (N = 3). E: SREBP1c induction by 4β-HC is stereospecific. Huh7 cells were treated with 4β-HC or an enantiomer of 4β-HC (ent-4HC) for 24 h in the indicated concentration (N = 3). Bars are the mean + SD. Statistical significance calculated by one-way ANOVA. ∗*P* < 0.05, ∗∗*P* < 0.01, ∗∗∗*P* < 0.001, ∗∗∗∗*P* < 0.0001. NS, not significant; 4β-HC, 4β-hydroxycholesterol.

To identify oxysterol ligands that could promote SREBP1c expression, we treated liver carcinoma–derived Huh7 cells with a panel of oxysterols selected among the most abundant in the bloodstream, including 4β-, 7β-, 19-, 20-, 24(S)-, 25-, and 27-HC. By quantitative PCR, several oxysterols previously identified as LXR activators, including 4β-HC, 7β-HC, 24(S)-HC and 25-HC, induced the expression of a canonical LXR target gene, *ABCA1*, with variable potency (Fig. 1A). In contrast, 4β-HC was the only oxysterol to induce significant upregulation of the SREBP1c transcript (Fig. 1B). A dose-response comparison between 4β-HC and 24(S)-HC showed that 24(S)-HC is a more potent activator than 4β-HC toward *ABCA1* (Fig. 1C) and another canonical LXR gene target, *ABCG1* (Fig. 1D). Conversely, 4β-HC activated *SREBP1c*, more potently than 24(S)-HC (Fig. 1E). 4β-HC–mediated induction of the *SREBP1c* gene was enantioselective, as the nonnatural enantiomer of 4β-HC (ent-4HC) was unable to induce SREBP1c mRNA expression even at the highest concentration used (20 μM) (Fig. 1F). These data suggest that *SREBP1c* induction depends on unique structural features of 4β-HC.

### 4β-HC induces expression and activation of SREBP1 but not SREBP2

Oxysterols such as 25- and 27-HC suppress SREBP1 and SREBP2 activation by blocking their trafficking to the Golgi, where proteolytic processing of the SREBPs to the mature nuclear form occurs (26, 27).

**Fig. 2:**
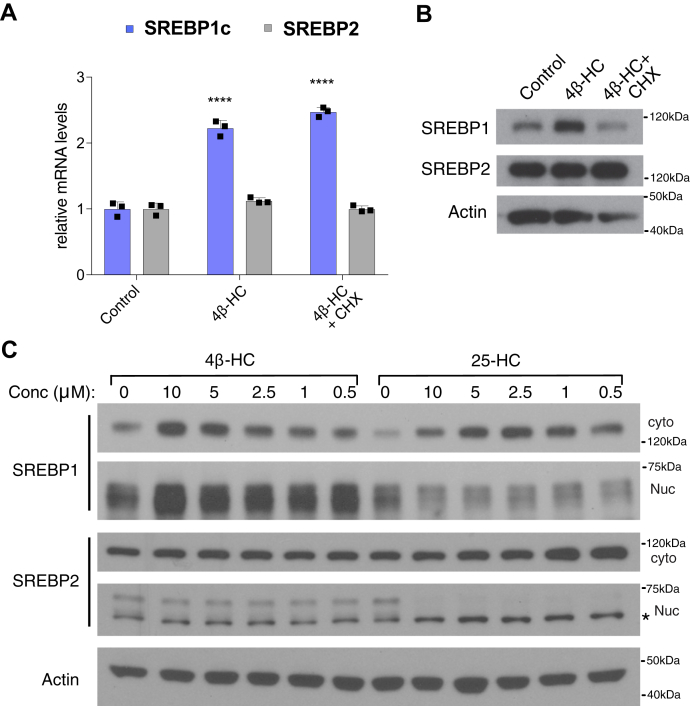
4β-HC induces expression and activation of SREBP1 but not SREBP2. A: 4β-HC increases SREBP1 protein expression. Huh7 cells were treated with 20 μM 4β-HC and a translation inhibitor, cycloheximide (CHX), for 4 h followed by measurement of SREBP1 and SREBP2 mRNA (N = 3) and (B) protein level (N = 1). C: 4β-HC increases SREBP1 cytosolic and nuclear levels while not affecting SREBP2. Huh7 cells were treated with 4β-HC or 25-HC for 24 h followed cytosolic-nuclear fractionation to measure protein level of SREBP1 and SREBP2 cytoplasmic and nuclear levels (N = 1). Asterisk denotes unspecific band in SREBP2 nuclear blot. Bars are the mean + SD. Statistical significance calculated by one-way ANOVA. ∗∗∗∗*P* < 0.0001. Cyto, cytosolic; Nuc, nuclear; 4β-HC, 4β-hydroxycholesterol.

In contrast to these oxysterols, 4β-HC significantly increased SREBP1c mRNA levels (Fig. 2A) and protein levels in a cycloheximide-sensitive manner (Fig. 2B). However, 4β-HC did not increase either mRNA or protein levels of SREBP2 (Fig. 2A, B). In keeping with the increased total levels of SREBP1c, 4β-HC increases both cytosolic and nuclear forms of SREBP1 in a dose-dependent manner, whereas levels of cytoplasmic or nuclear SREBP2 protein levels did not change (Fig. 2C). Consistent with previous reports (26, 27) and in contrast to 4β-HC, 25-HC reduced the nuclear forms of both SREBP1 and SREBP2, thereby causing the accumulation of the unprocessed cytoplasmic form of both proteins but without transcriptional upregulation (Fig. 1B).

These data suggest that, unlike other oxysterols that function as inhibitors of both SREBP1c and SREBP2, 4β-HC is a specific inducer of SREBP1c expression and activation.

### 4β-HC induce lipogenic programs through the LXRs

**Fig. 3:**
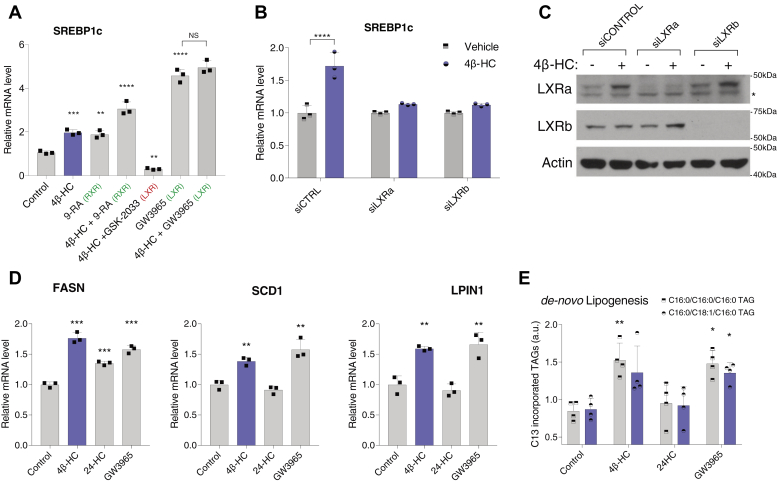
4β-HC induces lipogenic programs through the LXRs. A: 4β-HC interacts with LXR and RXR agonists and antagonists like an LXR ligand. Huh7 cells were treated with 20 μM 4β-HC, RXR agonist, 9-cis-retinoic acid (9-RA), LXR antagonist (GSK-2033), and LXR agonist (GW3965). For convenience, agonists are marked in green and antagonists are marked in red (N = 3). B: LXRα and LXRβ are required for SREBP1c induction by 4β-HC in Huh7 cells. Knockdown of LXRα or LXRβ by siRNA for 72 h followed by treatment with 5 μM 4β-HC for 24 h followed by RT-PCR of SREBP1c. (N = 3). C: Knockdown efficiency was evaluated by measurement of LXRα and LXRβ protein levels (N = 3). D: 4β-HC induction of lipogenic genes. Huh7 cells were treated for 24 h with 4β-HC, 24-HC, or LXR agonist (GW3965) followed by mRNA measurement of fatty acid synthase (FASN), stearoyl-CoA desaturase 1 (SCD1), and lipin1 (LPIN1) (N = 3). E: 4β-HC increases de novo lipogenesis. Huh7 cells were treated for 24 h with 5 μM 4β-HC, 24-HC, or LXR agonist (GW3965) with media containing C13 glucose followed by lipid extraction. C13 incorporation into TAGs was measured via LC/MS (N = 5). The asterisk denotes an unspecific band in the LXRα blot. Bars are the mean + SD. Statistical significance was calculated by one-way ANOVA. ∗*P* < 0.05, ∗∗*P* < 0.01, ∗∗∗*P* < 0.001, ∗∗∗∗*P* < 0.0001. 4β-HC, 4β-hydroxycholesterol; 24-HC, 24-hydroxycholesterol.

Along with other oxysterols, 4β-HC was previously shown to activate LXRα-dependent transcription in luciferase assays in vitro, supporting its role as a putative LXR ligand (4, 28). In turn, the LXR transcriptionally activates SREBP1c by directly binding to its promoter region (6). Combining these observations, we thus hypothesized that 4β-HC may transcriptionally activate SREBP1c and its downstream lipogenic programs via the LXR. Consistent with this possibility, cotreating cells with 4β-HC together with an LXR antagonist (GSK-2033) abolished 4β-HC–dependent induction of SREBP1c gene expression (Fig. 3A).

The effect of 4β-HC on SREBP1c induction was additive with an RXR ligand, 9-*cis*-retinoic acid. Moreover, coincubation of 4β-HC with the LXR agonist, GW3965, used at concentrations that activate the LXR maximally, caused no additional increase in SREBP1c expression over GW3965 alone (Fig. 3A). siRNA-mediated knockdown of either the LXRα or LXRβ (both of which are expressed in Huh7 cells) largely abolished 4β-HC–dependent SREBP1c mRNA expression (Fig. 3B). Interestingly, we noticed that 4β-HC treatment increased LXRα protein levels, a stabilizing effect observed for other established LXR ligands (29) (Fig. 3C). Together, and combined with previous reports these data support the hypothesis that 4β-HC induces SREBP1c gene expression by acting as an LXR agonist.

We next compared the ability of 4β-HC to induce SREBP1c-dependent lipogenic programs with that of the LXR agonist, GW3965. Fatty acid synthase (FASN), Stearoyl-CoA desaturase (SCD1) and Lipin1 (LPIN1) are validated SREBP1c downstream targets in Huh7 cells (30, 31). Treatment with either 4β-HC or GW3965 significantly increases the expression of these genes (Fig. 3D). In contrast, 24-HC, another putative LXR ligand that failed to induce SREBP1c in our hands (Fig. 1B, E), had minimal or no effect on these SREBP1c target genes (Fig. 3D).

Previous work had shown that GW3965 induces FASN to a greater extent than the 1.6-fold we observed in Huh7 (32). Huh7, a hepatocellular carcinoma line, is known to hyperactivated DNL to supply membranal lipids required for rapid division and growth (33, 34). We speculate that the modest increase in FASN by GW3965 or 4β-HC is due to already elevated baseline expression that cannot be increased much further. To further substantiate the prolipogenic effect of 4β-HC, we directly measured DNL by C13 incorporation into triglycerides using LC/MS. Similarly to lipogenic gene induction, both GW3965 and 4β-HC had a modest but statistically significant 1.5-fold increase in C13-labeled C16:C16:C16 TAG, or trending toward significance for C16:C18:C16 TAG, whereas 24-HC caused no significant change (Fig. 3E). Combined, these data suggest that the prolipogenic action of 4β-HC is comparable, in mechanism and potency, to known LXR agonists.

### 4β-HC induces lipid-droplet formation and triglyceride accumulation

**Fig. 4:**
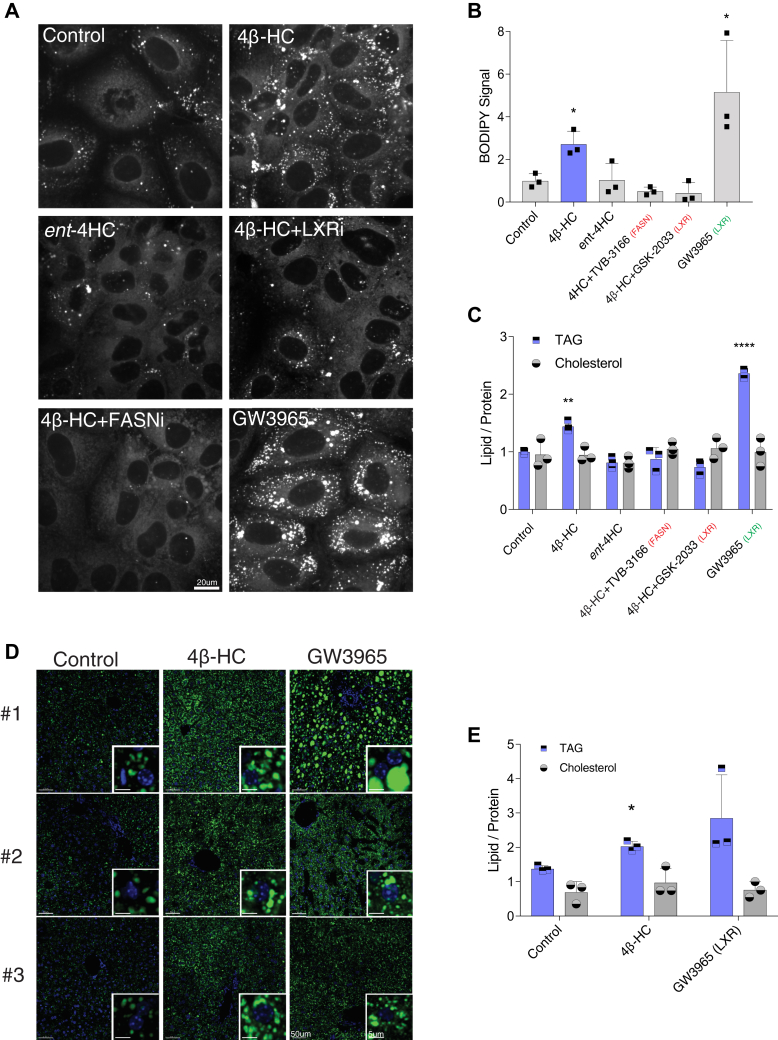
4β-HC induces lipid-droplet formation and triglyceride accumulation. A: 4β-HC increases the lipid droplet size and number. Huh7 cells were treated with 5 μM 4β-HC with indicated drugs for 72 h followed staining with lipid droplet dye, BODIPY 493/503, and visualization by confocal microscopy and (B) quantified using ImageJ (N = 3). C: 4β-HC increases triglycerides (TAG) levels. Huh7 cells were treat as (C) followed by measurement of triglycerides, total cholesterol, and protein levels using commercial kits (N = 3). D: 4β-HC increases lipid droplet in the mouse liver. Mice were fed normal chew with either vehicle 50 mg/kg/day 4β-HC or 10 mg/kg/day GW3965 for 5 days. Liver samples were fixed and stained with BODIPY 493/503 and DAPI to observe lipid droplet and nuclei ultrastructure (N = 3). E: 4β-HC increases triglycerides (TAG) levels in the mouse liver treated as above, followed by measurement of triglycerides, total cholesterol, and protein levels using commercial kits (N = 3). For convenience, agonists are marked in green and antagonists are marked in red. Bars are the mean + SD. Statistical significance calculated by one-way ANOVA.∗*P* < 0.05, ∗∗*P* < 0.01, ∗∗∗∗*P* < 0.0001. ent-4HC, stereo enantiomer-4HC, FASNi, TVB-3166; LXRi, GSK-2033; 4β-HC, 4β-hydroxycholesterol.

In keeping with the ability of 4β-HC to upregulate fatty acid biosynthetic genes via SREBP1c, treating Huh7 cells with 4β-HC (but not with its unnatural enantiomer, *ent*-4HC) for 72 h resulted in marked accumulation of lipid droplets (LDs), as revealed by staining with the lipophilic dye BODIPY 493/503 (Fig. 4A, B). LD accumulation induced by 4β-HC was suppressed by simultaneous treatment with a FASN inhibitor, TVB-3166, or with the LXR inhibitor GSK-2033. Measurement of triglyceride content in cell extracts confirmed the ability of 4β-HC to induce triglyceride accumulation, albeit with lower potency than the LXR agonist GW3965, whereas cholesterol levels remained unchanged (Fig. 4C). Consistent with the BODIPY staining, both LXR and FASN inhibitors hindered 4β-HC–induced triglyceride accumulation (Fig. 4C). Moreover, as seen with SREBP1c induction, the *ent*-4HC failed to induce triglyceride accumulation (Fig. 4C). Thus, 4β-HC is sufficient to induce the formation of triglyceride-containing LDs in an LXR- and FASN-dependent manner in cell culture.

Next, we tested the effect of 4β-HC on in vivo lipogenesis by feeding mice a normal diet supplemented with either 4β-HC or GW3965. After 7 days, the livers were harvested, LDs were assessed by BODIPY staining, and the liver lipid content (normalized to protein mass) was measured. Consistent with the results in Huh7 cells, 4β-HC significantly increased the size and number of LDs in liver sections (Fig. 4D) and liver triglyceride content (Fig. 4E), albeit with lower potency than the synthetic LXR agonist, GW3965. Collectively, these data suggest that 4β-HC is a prolipogenic factor that can increase liver lipid content in vivo.

### 4β-HC acts in parallel to insulin-PI3K signaling to drive SREBP1c expression

Insulin is a key hormone that drives SREBP1c transcription, proteolytic processing, and DNL in the postprandial state. Insulin regulates SREBP1c transcription via poorly understood mechanisms, which include AKT-dependent transcriptional downregulation of Insig-2a, the ER-retention factor that blocks translocation of SREBP cleavage-activating protein-SREBP1c to the Golgi (35, 36). The LXR was shown to be required for insulin-dependent activation on SREBP1c (37), but whether and how insulin activates LXR is not understood.

**Fig. 5:**
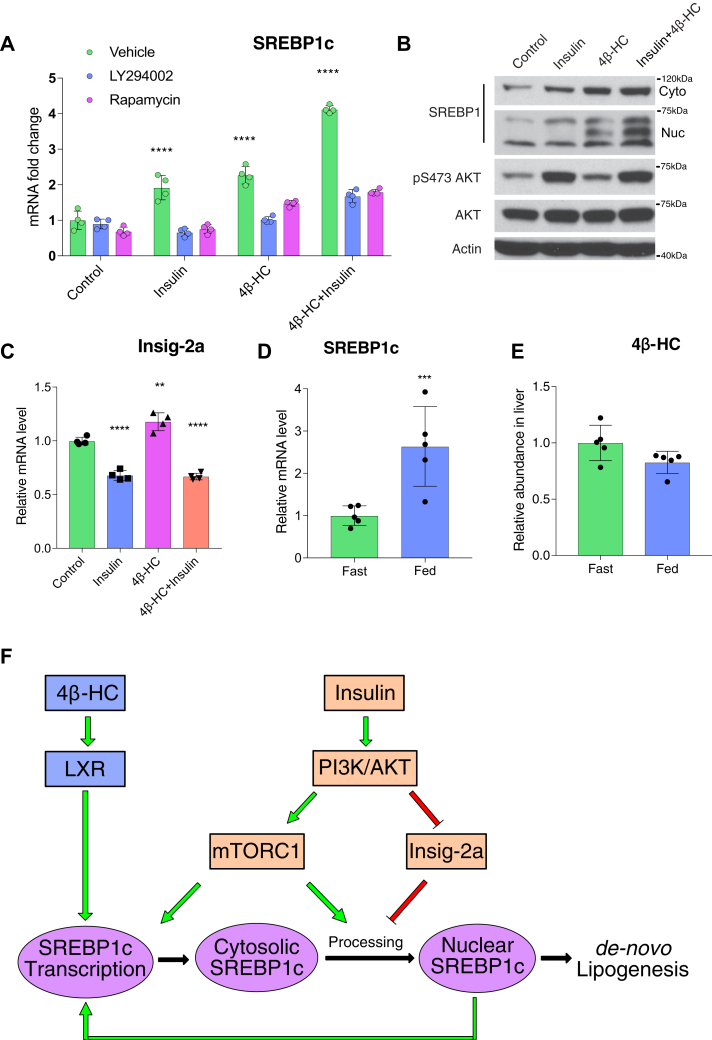
4β-HC acts in parallel to insulin-PI3K signaling to drive SREBP1c expression. A: SREBP1c transcription is additive by 4β-HC and insulin. Primary hepatocytes were treated overnight with vehicle or 5 μM 4β-HC followed with 6 h stimulation with combinations of insulin, PI3K inhibitor (LY294002), or rapamycin. The SREBP1c mRNA level was measured by RT-PCR (N = 4). B: 4β-HC and insulin have an additive effect on SREBP1c expression and nuclear processing. Primary hepatocytes were treated overnight with vehicle or 4β-HC followed by addition of insulin for 40 min. Proteins were extracted and SREBP1 and AKT protein levels were measured (N = 2). C: Primary hepatocytes were treated with 4β-HC and insulin as described in (A) followed by RT-PCR measurement of Insig-2a mRNA level (N = 4). D: Insulin does not induce 4β-HC synthesis. Mice were fasted for 16 h and then refed for 4 h, followed by liver extraction and RT-PCR for SREBP1c mRNA level (N = 4) and (E) 4β-HC levels by MS (N = 5). F: Model; the 4β-HC-LXR pathway acts in parallel to the insulin-PI3K pathway to drive SREBP1c expression in an additive fashion. ∗∗*P* < 0.01, ∗∗∗*P* < 0.001, ∗∗∗∗*P* < 0.0001.

To interrogate the relationship between 4β-HC and insulin signaling in driving SREBP1c transcription and processing, we used an insulin-responsive primary mouse hepatocyte (38). In these cells, stimulation with either 4β-HC or insulin alone increased the mRNA levels of SREBP1c, while combined 4β-HC and insulin increased SREBP1c mRNA levels additively [as previously shown for LXR agonists (37)] (Fig. 5A). Interestingly, treatment with PI3K or mTORC1 inhibitors abolished SREBP1c induction by both insulin and 4β-HC (Fig. 5A), raising the possibility of a ‘coincidence detection’ model, in which a minimal amount of both insulin-PI3K-mTORC1 and 4β-HC signaling must be present for SREBP1c induction to occur.

Similar to their effects on transcriptional induction, insulin and 4β-HC stimulated proteolytic processing of SREBP1c in an additive manner (Fig. 5B). 4β-HC did not affect AKT phosphorylation significantly, suggesting that insulin-PI3K-AKT and 4β-HC signaling act in parallel and converge at the level of the SREBP1 gene promoter (Fig. 5B and supplemental Fig. 1A).

Consistent with previous reports, we also detected a marked decrease in Insig-2a mRNA in insulin-stimulated hepatocytes (Fig. 5C). In contrast, 4β-HC caused a mild increase of Insig-2a mRNA levels, and combined insulin and 4β-HC was similar to insulin alone, suggesting Insig-2a downregulation is not required for 4β-HC–dependent SREBP1c activation (Fig. 5C).

To further probe possible connections between insulin-PI3K and 4β-HC-LXR signaling, we tested whether insulin signaling promotes 4β-HC synthesis. Previous reports had shown that in humans, 4β-HC has a very slow kinetics, with an extremely long half-life in plasma (60 h) (39). Pharmacological induction of the main 4β-HC–synthesizing enzyme, cytochrome P450 3A, doubles 4β-HC concentration in human plasma in 8 days (40), a very different pattern from insulin, which peaks within 1–2 h after a meal and drops in between. On the other hand, in vitro work in primary rat hepatocytes led to the hypothesis that insulin signaling may produce an unknown LXR ligand that, in turn, induces SREBP1c (37). To test the possibility of insulin-dependent 4β-HC production, we compared 4β-HC levels in the liver of mice that were either fasted or refed. Although mice that were refed showed significant induction of SREBP1c transcription, consistent with SREBP1c regulation by insulin (Fig. 5D), the levels of 4β-HC did not increase accordingly in the liver (Fig. 5E) or serum (Supplemental Fig. 1B). Collectively, these data suggest that insulin does not induce 4β-HC production according to fasting/feeding cycles and that 4β-HC most likely acts in parallel to insulin-PI3K signaling in driving SREBP1c transcription and SREBP1c-dependent DNL (Fig. 5F).

## Discussion

Here we identify 4β-HC as a unique oxysterol that activates SREBP1c expression and promotes lipogenic gene programs, resulting in induction of fatty acid biosynthesis and cellular accumulation of triglycerides in LDs both in cell culture and in vivo. Our results are most consistent with a model in which 4β-HC acts in parallel to insulin-PI3K-mTOR signaling, and the two pathways have additive effects on SREBP1c activation. A simple mechanism that explains the additive effect is that the SREBP1c promoter contains both an LXR-binding element and an SREBP-binding element and that transcription can be stimulated by the two transcription factors independently (41). However, the observation that inhibition of PI3K-AKT signaling also blunts 4β-HC–dependent SREBP1 induction (Fig. 5A) points to a possible ‘coincidence detection’ model, where at least some signaling by one input (i.e., insulin) has to be present for the other input (4β-HC) to be effective, and vice versa. From a temporal standpoint, 4β-HC kinetics suggest that it stimulates SREBP1c expression in a chronic manner, whereas insulin acts acutely in the postprandial state.

A recent publication by Salonurmi *et al*. (42) showed that 4β-HC induces cholesterol efflux from peripheral mononuclear cells in vivo via transcriptional upregulation of *ABCA1* and concomitant suppression of influx transporters. In our hands, 4β-HC did not induce *ABCA1* expression in primary hepatocytes (supplemental Fig. 1C), whereas its induction was observed in Huh7 cells. Thus, 4β-HC–dependent regulation of cholesterol efflux versus DNL may be cell type specific and tied to different physiological settings.

Several groups using different animal models (mice, rats, rabbits, and swine) had all observed that 4β-HC levels increase when animals are fed a high cholesterol diet (43, 44, 45, 46), whereas a high-fat but with low-cholesterol diet reduces 4β-HC levels in mice (47). Dietary cholesterol was shown to increase *SREBP1c* expression in an LXR-dependent manner (6, 9). Furthermore, genetically disrupting hepatic cholesterol synthesis through *SREBP2* KO also causes *SREBP1c* downregulation, which can be rescued by an LXR agonist (48). This study also determined that 4β-HC levels are decreases in young SREBP2-null mice, defining a correlation between SREBP2-dependent cholesterol synthesis, 4β-HC levels, and *SREBP1c* expression. Together with this published literature, our results strongly suggest that 4β-HC may be the cholesterol-derived molecule that induces *SREBP1c* activation via the LXR.

An important question is why 4β-HC is the sole oxysterol ligand of LXRs to activate SREBP1 expression in our hands. Several possibilities can be envisioned. The LXR-RXR heterodimer can recruit coactivators (PGC-1α, TRRAP, ACS-2, p300, SRC-1) and corepressors (NCoR, SMRT) to the promoters of target genes in a ligand-dependent manner (49, 50, 51, 52, 53), but whether all LXR ligands are equally effective in recruiting specific combinations of cofactors is unclear. Supporting this model was an observation in macrophages that the ability of the LXR to recruit RNA polymerase II to *SREBP1c* promoter requires a specific LXR ligand, while recruitment of RNA polymerase II to the *ABCA1* promoter is more promiscuous (29). Thus, 4β-HC may be able to direct a unique set of coactivators and RNA polymerase II to the *SREBP1c* promoter, resulting in its activation.

Consistent with previous reports, the synthetic LXR agonist GW3965 was also able to trigger *SREBP1* expression (18, 19). Synthetic LXR agonists are generally more potent than natural LXR ligands, possibly reflecting higher affinity for the ligand-binding site of the LXR. By analogy, 4β-HC may bind to the LXR with higher affinity than other oxysterol ligands. In turn, higher affinity may translate into longer residence time on the *SREBP1c* promoter DNA, a possible prerequisite for its efficient activation.

Our data point to the importance of the enzyme that produces 4β-HC, Cyp3A4 (Cyp3A11 in mice) (54), as a crucial regulator of lipogenesis. Consistent with that, several groups have reported that increased Cyp3A4 expression by overexpressing its activator, pregnane X receptor, correlated with increases in lipogenic gene expression and liver triglyceride levels (55, 56). Conversely, decreased Cyp3A4 expression (57) or its pharmacological inhibition (58) was associated with lower lipogenic gene expression and liver triglyceride levels. Taken together, these data suggest that Cyp3A4 and 4β-HC may regulate diet-induced lipogenic genes and liver triglyceride levels.

From a more clinical perspective, 4β-HC might have an aggravating effect on the development of NAFLD. NAFLD is characterized by elevated liver triglycerides not due to alcohol consumption or any other known causes (59). Elevated triglyceride levels are associated with LXR and SREBP1c upregulation in NAFLD (60). Patients with NAFLD show a significant increase in 4β-HC plasma levels compared with healthy patients (61). Thus, it is plausible that elevated 4β-HC levels could be an unrecognized driver of triglyceride accumulation in NAFLD. It would be interesting to determine the effect of pharmacologic Cyp3A4 inhibition on disease progression in patients with NAFLD.

In conclusion, this work highlights a role for 4β-HC, which was long viewed as an ‘orphan’ oxysterol, in regulating lipid metabolism in the liver together with insulin. Future work, dissecting the role of 4β-HC in other organs and in different pathological settings, will provide a full picture on the function and significance of this highly abundant oxysterol.

## Materials and methods

### Materials

Reagents were purchased from the following sources. Antibodies used are as follows: SREBP1 (2A4, Santa Cruz Biotechnology), SREBP2 (30682, Abcam), LXRα (PP-PPZ0412-00, R&D systems), LXRβ (K8917, R&D systems), phospho-T308 AKT (C31E5E), AKT (11E7) (Cell Signaling Technology).

Drugs used are as follows: Cycloheximide (Cell Signaling Technology) was used at 10 μg/ml. 9-*cis*-retinoic acid (Sigma) was used at 50 μM. The LXR antagonist GSK-2033 (Axon Medchem) was used at 500 nM. The LXR agonist GW3965 (Fisher Scientific) was used at 500 nM. PI3K inhibitor, LY294002 (Cell Signaling Technology), was used at 10 μM. Rapamycin was used at 100 nM and received as a gift from David Sabatini. Methyl-beta-cyclodextrin was purchased from Sigma. All sterols except for custom-synthesized ent-4β-HC (see below) were purchased from Steraloids. C13 glucose was purchased from Cambridge Isotope Laboratories.

### Sterol: methyl-beta-cyclodextrin precomplexing

All sterols were made to 50 mM stocks in ethanol. To deliver the sterols to cells, 1.25 mM sterol was complexed with 25-mM methyl-beta-cyclodextrin and vortexed until the solution was clear. Sterols were added to the media in an indicated concentration and incubation time. Control samples were treated by adding the same volume of ethanol to methyl-beta-cyclodextrin, which then was delivered to cells in the same corresponding volume.

### Cell culture

Huh7 cells were maintained on DMEM (5 g/l glucose + glutamine, Gibco) supplemented with 10% FBS (VMR) and p/s (Gibco). Lipid-depleted serum (LDS) was made as described (62). For assays, on day one; 10^5^ cells were plates in 6 cm plates. On day 2, media was changed to 1% LDS and 1 g/l glucose DMEM. On day 3; plates were spiked with precomplexed sterols for indicated times, concentrations, and additional compounds.

Primary mouse hepatocytes were purchased from the UCSF liver center. The isolation protocol is based on the study by Li, Brown, and Goldstein (63) and adjusted in the following manner. Mice were fasted overnight before isolation. Hepatocytes were isolated by the perfusion protocol (64) and plated at density of 7 × 10^5^/well on 6-well collagen-coated plates (Corning) in DMEM supplemented with 10% FBS. Once cells adhere, the media was replaced to Medium 199 (GIBCO) containing 100 nM dexamethasone (Sigma), 100 nM 3,3,5-triiodo-L-thyronine (T3, Sigma), and Insulin-Transferrin-Selenium (Gibco). Next day, the same media was used without Insulin-transferrin-Selenium to assay insulin, 4β-HC, and inhibitors at indicated times and concentrations.

### Real-time PCR analysis for gene expression

RNA was extracted using the RNeasy kit (Qiagen). One microgram of RNA was reverse-transcribed using Super Script III (Invitrogen). Quantitative PCR was performed using Ssoadvanced (Bio-Rad) in StepOnePlus (ABI). The list of primers is in Table 1.

**Table 1:** RT-PCR primers

Gene	Species	Forward	Reverse
TBP	Human	TTGTACCGCAGCTGCAAAAT	TATATTCGGCGTTTCGGGCA
SREBP1c	Human	GCGCCTTGACAGGTGAAGTC	GCCAGGGAAGTCACTGTCTTG
FASN	Human	CTTCAAGGAGCAAGGCGTGA	ACTGGTACAACGAGCGGATG
SCD1	Human	TCTAGCTCCTATACCACCACCA	TCGTCTCCAACTTATCTCCTCC
ABCA1	Human	TGTTCGCGGCCCTCAT	CGAGATATGGTCCGGATTGC
ABCG1	Human	TGCAATCTTGTGCCATATTTGA	CCAGCCGACTGTTCTGATCA
LPIN1	Human	CCAGCCCAATGGAAACCTCC	AGGTGCATAGGGATAACTTCCTG
SREBP2	Human	GAGCTGGGTGGTCTGGAG	TTGCAGCATCTCGTCGATGT
SREBP1C	Mouse	CGGAAGCTGTCGGGGTAG	GTTGTTGATGAGCTGGAGCA
SREBP2	Mouse	GCGTTCTGGAGACCATGGA	ACAAAGTTGCTCTGAAAACAAATCA
INSIG-2A	Mouse	TGTGAGCTGGACTAGCTTGCT	CCTAAGCCGTAAAACAAAATG
TBP	Mouse	ACCCTTCACCAATGACTCCTATG	ATGATGACTGCAGCAAATCGC

### Protein extraction and Western blot

Cells were harvested with the RIPA buffer supplemented with Phosphatase inhibitor and protease inhibitor (10 mM Tris Cl (pH 8.0), 1 mM EDTA, 1% Triton X-100, 0.1% sodium deoxycholate, 0.1% SDS, 140 mM NaCl, 10 mM Na-PPi, 10 mM Na-Beta-glycerophosphate), sonicated with Bioruptor (Diagenode), and normalized using the BCA kit (Thermo Scientific).

### Knockdown using siRNA

siRNA ON-TARGET plus smart pool against LXRα (cat# L-003413-00-0005), LXRβ (cat# L-003412-02-0005), or nontargeted siRNA ON-TARGETplus Non-targeting Pool (cat# D-001810-10-05) was purchased from Dharmacon. Five micromolar siRNA was mixed with 5 μl Lipofectamine RNAiMAX (Life Technologies) in Opti-MEM (Gibco). siRNA is added to preplated Huh7 (10^5^ cells/6 cm plate) in regular media without penicillin streptomycin for 5 h followed by replacement to regular media for 72 h.

### LD microscopy

Huh7 were plated on a coverslip coated with fibronectin (Corning) and treated as indicated with sterols and drugs. Cells were fixed with paraformaldehyde and stained with 1 μg/ml BODIPY 493/503 for 1 h. Coverslips were mounted with VECTASHIELD with DAPI (Vector Laboratories) and imaged on a spinning disk confocal system (Andor Revolution on a Nikon Eclipse Ti microscope). The BODIPY signal was measured using ImageJ and normalized by the number of nuclei.

### Triglyceride and cholesterol measurements

Liver samples were powdered with a pestle and mortar and lysed in the RIPA buffer. Huh7 cells were also harvested in the RIPA buffer. Five microliters of the samples was used to measure triglyceride using Triglyceride Infinity (Thermo Fisher) or cholesterol using the Amplex red cholesterol measuring kit (Invitrogen) in a clear 96-well sample. The BCA kit (Thermo Scientific) was used for normalization of the protein level. Absorbance and fluorescence were measured by the PerkinElmer Envision Multilabel plate reader.

### C13 incorporation into triglycerides

Huh7 cells were seeded at 200K per 6-cm plates. The next day, DMEM media with glutamine, containing 5 mM C13 glucose (Cambridge Isotope Laboratories) and 1% LDS including oxysterols and the LXR agonist were added for 24 h. C12 glucose–treated plates were used as reference. Cells were washed twice with ice-cold PBS and scraped, and pellets were snap-frozen and kept in −80°C for later analysis. Lipid extraction and analysis by LC/MS was performed as described (65).

### Husbandry and diets

All mouse procedures were performed and approved under the University of California, Berkeley Animal Care and Use Committee. Ten-week-old C57BL/6J male mice were purchased from the Jackson Laboratory and housed for one week in our facility under standard conditions before experiments were performed. Free access to water and chow (Lab Diets, #3038) was provided throughout this acclimation period. Afterward, mice were placed on a diet with 50 mg/kg/day 4β-HC or LXR agonist GW3965 10 mg/kg/day for 7 days. Powdered 10% by kCal fat diet (Research Diets Inc. #D12450J) was used as the base of each treatment food, forming pellets that were dried overnight at room temperature in a laminar flow hood. After 7 days, mice were euthanized using CO_2_ and cervical dislocation.

### Cryosectioning and fluorescent histochemistry

Liver samples were fixed using 4% (v/v) paraformaldehyde overnight at 4°C. The next day, samples were cryopreserved using sterile-filtered 30% sucrose (w/v) in dulbecco’s phosphate buffered saline (DPBS) (Gibco, 14190-144). After 3 days, each sample was placed in a 1:1 30% sucrose:Neg-50 (Richard-Allan Scientific) solution and incubated overnight at 4°C. The samples were frozen on dry ice using undiluted Neg-50 at −50°C and stored at −80°C until sectioning. Sequential 20 μM thick sections were obtained from each sample using a Leica CM3050S cryostat.

For nuclei and LD labeling, sectioned tissue was washed three times at room temperature in DPBS for 5 min each. Afterward, DPBS containing 10 μM BODIPY (Invitrogen, #D3922) was placed on the samples and incubated for 30 min at room temperature in the dark. Next, the slides were washed with DPBS twice before incubating in DPBS containing 5 μg/ml DAPI (Invitrogen, D1306) for 10 min at room temperature in the dark. After DAPI staining, the slides were washed three times in DPBS for 5 min each before being mounted using SlowFade Diamond antifade (Invitrogen, #S36972) and sealing with nail polish overnight. Slides were imaged immediately using a Zeiss LSM710 confocal microscope. Images were developed using the IMARIS (Bitplane) image analysis software suite.

### Synthesis of *ent-4β-HC*

**Figure:**
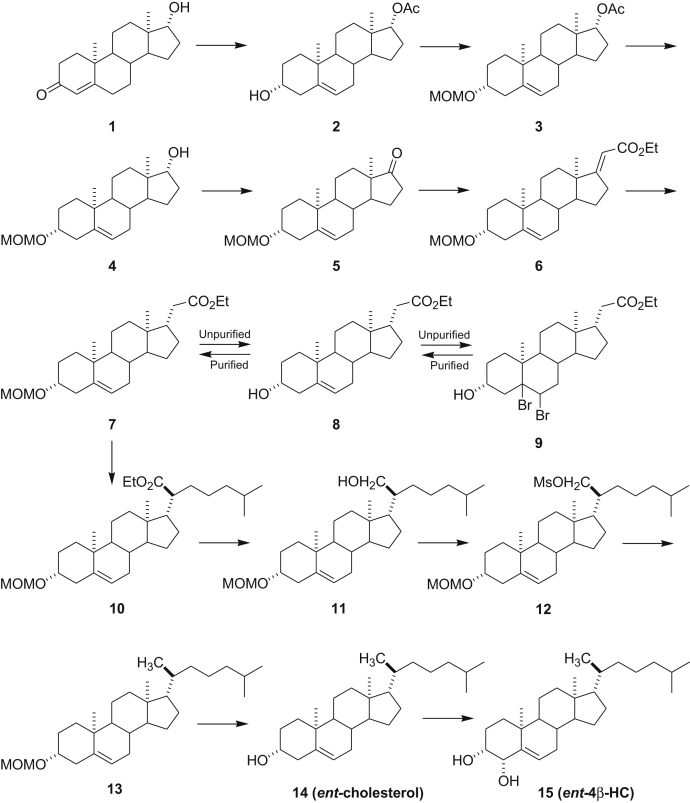

#### *ent-*steroid 2

*ent-*Testosterone (1) was prepared as described previously [(66); see also references therein]. To a solution of *ent*-testosterone (1, 3.8 g, 13.2 mmol) in acetic anhydride (80 ml) was added NaI (7.92 g, 52 mmol) and trimethylsilyl chloride (5.8 ml, 52 mmol) at 0°C under N_2_. After addition, the reaction was allowed to warm to room temperature for 2 h. The reaction was added to Et_3_N (40 ml) in diethyl ether (100 ml). The ether solution was washed with brine (50 ml × 4) and aqueous NaHCO_3_ (50 ml × 2) and dried over Na_2_SO_4_. After filtration, the solvent was removed under reduced pressure and the residue was purified by flash column chromatography (silica gel eluted with 25% ethyl acetate (EtOAc) in hexanes) to give *ent-*steroid 2 (3.05 g, 70%): ^1^H NMR (400 MHz, CDCl_3_) δ 5.33–5.32 (m, 1H), 4.60 (t, *J* = 8.3 Hz, 1H), 3.52–3.47 (m, 1H), 2.30–0.90 (m), 2.02 (s, 3H), 1.00 (s, 3H), 0.79 (s, 3H); ^13^C NMR (100 MHz, CDCl_3_) δ 171.2, 140.9, 121.1, 82.7, 71.5, 51.0, 50.0, 42.3, 42.2, 37.2, 36.7, 36.5, 31.6, 31.5, 31.4, 27.4, 23.5, 21.1, 20.5, 19.3, 11.8.

#### *ent-*steroid 3

*ent-*Steroid 2 (3.05 g, 4.04 mmol) was dissolved in CH_2_Cl_2_ (50 ml) and cooled to 0°C. (*i-*Pr)_2_EtN (3.0 ml) and ClCH_2_OMe (1.35 ml, 18.0 mmol) were added, and the reaction was stirred at room temperature for 16 h. The reaction was made basic by adding aqueous NaHCO_3_ solution, and the product was extracted into CH_2_Cl_2_. The combined extracts were washed with brine, dried over Na_2_SO_4_, filtered, and solvent removed to give a viscous liquid that was purified by flash column chromatography (silica gel eluted with 10% EtOAc in hexanes) to give *ent-*steroid 3 as a colorless liquid (2.65 g, 77%): ^1^H NMR (400 MHz, CDCl_3_) δ 5.33–5.32 (m, 1H), 4.65 (s, 2H), 4.59 (t, *J* = 8.2 Hz, 1H), 3.39–3.35 (m, 1H), 3.34 (s, 3H), 2.35–0.89 (m), 2.01 (s, 3H), 0.99 (s, 3H), 0.78 (s, 3H); ^13^C NMR (100 MHz, CDCl_3_) δ 171.0, 140.7, 121.2, 94.6, 82.6, 76.7, 55.0, 50.9, 50.0, 42.3, 39.4, 37.1, 36.7, 31.6, 31.4, 28.8, 27.4, 23.5, 21.0, 20.4, 19.3, 11.8.

#### *ent-*steroid 4

To a solution of *ent-*steroid 3 (2.65 g, 7.05 mmol) in methanol (60 ml), K_2_CO_3_ (4.0 g) was added at room temperature. The mixture was refluxed for 16 h. Methanol was removed under reduced pressure, and the residue was purified by flash column chromatography (silica gel eluted with 25% EtOAc in hexanes) to give *ent-*steroid 4 (2.31 g, 99%): ^1^H NMR (400 MHz, CDCl_3_) δ 5.32–5.30 (m, 1H), 4.64 (s, 2H), 3.61 ( t, *J* = 8.6 Hz, 1H), 3.40–3.34 (m, 1H), 3.33 (s, 3H), 2.31–0.87 (m), 0.95 (s, 3H), 0.72 (s, 3H); ^13^C NMR (100 MHz, CDCl_3_) δ 140.7, 121.3, 94.5, 81.6, 76.7, 55.0, 51.2, 50.2, 42.6, 39.4, 37.2, 36.7, 36.5, 31.8, 31.4, 30.3, 28.8, 23.3, 20.5, 19.3, 10.9.

#### *ent-*steroid 5

To a solution of *ent-*steroid 4 (1.5 g, 4.54 mmol) in CH_2_Cl_2_ (60 ml), Dess–Martin periodinane (2.5 g, 6 mmol) was added at room temperature. After 1 h, water (50 ml) was added, the product was extracted into CH_2_Cl_2_ (150 ml × 3), and the combined extracts were washed with brine (50 ml × 2). The organic layer was dried over Na_2_SO_4_ and filtered and the solvents were removed. The residue was purified by flash column chromatography (silica gel eluted with 10% EtOAc in hexanes) to give *ent-*steroid 5 (1.5 g, 100%): ^1^H NMR (400 MHz, CDCl_3_) δ 5.39–5.38 (m, 1H), 4.68 (s, 2H), 3.45–3.38 (m, 1H), 3.37 (s, 3H), 2.49–0.98 (m), 1.03 (s, 3H), 0.88 (s, 3H); ^13^C NMR (100 MHz, CDCl_3_) δ 221.0, 140.9, 120.9, 94.7, 76.7, 55.1, 51.7, 50.2, 47.5, 39.5, 37.1, 36.8, 35.8, 31.4, 31.3, 30.8, 28.8, 21.8, 20.3, 19.3, 13.5.

#### *ent-*steroid 6

A solution of freshly prepared sodium ethoxide (sodium 0.4 g, 15 mmol dissolved in ethanol 15 ml) was added dropwise slowly to a solution of *ent-*steroid 5 (1.5 g, 4.54 mmol) and triethyl phosphonoacetate (3.44 g, 15 mmol) in anhydrous ethanol (25 ml) under N_2_ while stirring at 35–40°C. After addition, the reaction was refluxed for 16 h. After cooling to room temperature, the ethanol was removed and the residue was dissolved in ether, which was washed with water, dried over Na_2_SO_4_, and filtered. The solvent was removed, and the residue was purified by flash column chromatography (silica gel eluted with 10% EtOAc in hexanes) to give *ent-*steroid 6 (1.68 g, 87%): ^1^H NMR (400 MHz, CDCl_3_) δ 5.52 (s, 1H), 5.35–5.34 (m, 1H), 4.66 (s, 2H), 4.15–4.09 (m, 2H), 3.43–3.33 (m, 1H), 3.35 (s, 3H), 2.84–2.79 (m, 2H), 2.36–0.93 (m), 1.01 (s, 3H), 0.82 (s, 3H); ^13^C NMR (100 MHz, CDCl_3_) δ 176.1, 167.3, 140.7, 121.3, 108.6, 94.6, 76.7, 59.4, 55.1, 53.7, 50.2, 46.0, 39.5, 37.2, 36.8, 35.1, 31.6, 31.5, 30.4, 28.8, 24.4, 20.9, 19.3, 18.2, 14.3.

The reaction sequence reported below that converts *ent-*steroid 6 into *ent-*steroid 16 (*ent-*VP1-001) is based on that reported previously for the preparation of the natural stereoisomer of *ent-*steroid 16 (67).

#### Unpurified *ent-*steroid 7

To a solution of *ent-*steroid 6 (1.4 g, 3.48 mmol) in EtOAc (150 ml), PtO_2_ (15 mg) was added at room temperature. Hydrogenation was carried out under 20 psi for 6 h. The solvent was removed, and the residue was purified by flash column chromatography (silica gel eluted with 10% EtOAc in hexanes) to give unpurified *ent-*steroid 7 (1.4 g, 100%): ^1^H NMR δ 4.63–4.60 (m, 1H), 4.08–4.03 (m, 2H), 3.48–3.32 (m, 1H), 3.31 (s, 3H), 2.34–0.57 (m), 0.76 (s, 3H), 0.54 (s, 3H); ^13^C NMR δ 176.1, 140.7, 121.3, 94.4, 76.2, 60.0, 55.3, 55.0, 54.5, 46.9, 44.9, 42.1, 37.4, 37.0, 35.6, 35.5, 35.3, 35.2, 32.1, 28.7, 28.1, 24.4, 20.9, 14.2, 12.5.

Unpurified *ent-*steroid 7 contains minor amounts of the *ent-*steroid in which the Δ^5^ double bond has been hydrogenated. This saturated *ent-*steroid could not be removed easily by chromatography on silica gel. To separate the two compounds chromatographically, *ent-*steroid 7 was converted first into *ent-*steroid 8 and then into *ent-*steroid 9, which is easily purified. *ent-*Steroid 9 was then converted back via *ent-*steroid 8 into *ent-*steroid 7 and then subsequently into *ent-*steroid 10.

#### Unpurified *ent-*steroid 8

Acetyl chloride (2 ml) was slowly added to unpurified hydrogenation product *ent-*steroid 7 (1.4 g, 3.48 mmol) in ethanol (30 ml) at room temperature. After 2 h, water was added and the product was extracted into CH_2_Cl_2_ (100 ml × 2). The combined extracts were dried over Na_2_SO_4_ and filtered, and the solvent was removed under reduced pressure. The residue was purified by flash column chromatography (silica gel eluted with 25% EtOAc in hexanes) to give unpurified *ent-*steroid 8 (1.2 g): ^1^H NMR (400 MHz, CDCl_3_) δ 5.35–5.34 (m, 1H), 4.13–4.07 (m, 2H), 3.55–3.47 (m, 1H), 2.38–0.81 (m), 1.10 (s, 3H), 0.61 (s, 3H); ^13^C NMR (100 MHz, CDCl_3_) δ 173.9, 140.8, 121.5, 71.6, 60.1, 55.5, 50.3, 46.8, 42.2, 41.9, 37.3, 37.2, 36.5, 35.2, 31.9, 31.8, 31.6, 28.1, 24.5, 20.8, 19.4, 14.2, 12.4.

#### *ent-*steroid 9

To a solution of unpurified *ent-*steroid 8 (1.2 g, 3.33 mmol) in diethyl ether (100 ml) and acetic acid (5 ml), Br_2_ in HOAc (3 ml) was added slowly until brown color persisted. After 5 min, aqueous Na_2_S_2_O_3_ was added and the reaction became colorless. EtOAc (100 ml) was added, and the EtOAc solution was washed with aqueous NaHCO_3_ (50 ml × 2), brine (50 ml), and dried over anhydrous Na_2_SO_4_. After filtration, the solvent was removed under reduced pressure, and the residue was purified by flash column chromatography (silica gel eluted with 20% EtOAc in hexanes) to give *ent-*steroid 9 (1.4 g, 81%): ^1^H NMR (400 MHz, CDCl_3_) δ 4.82–4.81 (m, 1H), 4.44–4.37 (m, 1H), 4.12–4.06 (m, 2H), 2.72–1.08 (m), 1.43 (s, 3H), 0.62 (s, 3H); ^13^C NMR (100 MHz, CDCl_3_) δ 173.8, 89.6, 68.9, 60.1, 56.0, 54.0, 47.6, 46.6, 45.6, 42.2, 42.0, 37.2, 37.0, 36.7, 35.2, 30.9, 30.1, 28.0, 24.2, 21.0, 20.3, 14.2, 12.7.

#### Purified *ent-*steroid 8

Zinc dust (6.0 g) was added to a solution of *ent-*steroid 9 (1.4 g, 2.7 mmol) in HOAc (20 ml) and EtOAc (30 ml) at room temperature. After 16 h, the mixture was filtered through Celite and washed with EtOAc (200 ml). The solvent was removed under reduced pressure, and the residue was purified by flash column chromatography (silica gel eluted with 25% EtOAc in hexanes) to give purified *ent-*steroid 8 (925 mg, 95%): ^1^H NMR (400 MHz, CDCl_3_) δ 5.26–5.25 (m, 1H), 4.06–4.01 (m, 2H), 3.85 (s, br, 1H), 3.47–3.40 (m, 1H), 2.31–0.73 (m), 0.93 (s, 3H), 0.54 (s, 3H); ^13^C NMR (100 MHz, CDCl_3_) δ 173.8, 140.7, 121.1, 71.2, 60.0, 55.4, 50.1, 46.6, 41.9, 41.7, 37.1, 37.0, 36.3, 35.0, 31.7, 31.7, 31.2, 27.9, 24.3, 20.6, 19.2, 14.0, 12.2.

#### Purified *ent-*steroid 7

Purified *ent-*steroid 8 (925 mg, 2.57 mmol) was dissolved in CH_2_Cl_2_ (20 ml) and cooled to 0°C. (*i-*Pr)_2_EtN (1.3 ml, 7.5 mmol) and ClCH_2_OMe (0.45 ml, 6.0 mmol) were added, and the reaction was stirred at room temperature for 16 h. The reaction mixture was made basic by adding aqueous saturated NaHCO_3_ solution and the product extracted into CH_2_Cl_2_. The combined extracts were washed with brine, dried over anhydrous Na_2_SO_4_, and the solvent removed to give a viscous liquid that was purified by flash column chromatography (silica gel eluted with 20% EtOAc in hexanes) to give purified *ent-*steroid 7 as a colorless liquid (1.02 g, 98%): ^1^H NMR (400 MHz, CDCl_3_) δ 5.34–5.33 (m, 1H), 4.67 (s, 2H), 4.12 (q, *J* = 7.0 Hz, 2H), 3.42–3.36 (m, 1H), 3.35 (s, 3H), 2.37–0.80 (m), 1.00 (s, 3H), 0.60 (s, 3H); ^13^C NMR (CDCl_3_) δ 173.8, 140.7, 121.5, 94.6, 76.8, 60.0, 55.5, 55.1, 50.3, 46.7, 41.9, 39.5, 37.2, 37.1, 36.7, 35.2, 31.9, 31.8, 28.9, 28.1, 24.5, 20.7, 19.3, 14.2, 12.3.

#### *ent-*steroid 10

To a solution of the *ent-*steroid 7 (202 mg, 0.5 mmol) in tetrahydrofuran (THF) (10 ml), lithium diisopropylamide (0.75 ml, 2.0 M in THF, 1.5 mmol) and HMPA (0.29 ml, 1.65 mmol) were added at –78°C. After 1 h, 1-bromo-4-methylpentane (0.44 ml, 3 mmol) was added. After addition, the reaction was warmed to room temperature for 16 h. Aqueous NH_4_Cl was added and extracted with EtOAc (100 ml × 2), and the combined extracts were dried over anhydrous Na_2_SO_4_. The solvent was removed under reduced pressure, and the residue was purified by flash column chromatography (silica gel eluted with 20% EtOAc in hexanes) to give *ent-*steroid 10 (236 mg, 97%): ^1^H NMR (400 MHz, CDCl_3_) δ 5.34–5.33 (m, 1H), 4.67 (s, 2H), 4.13–4.08 (q, *J* = 7.4 Hz, 2H), 3.41–3.37 (m, 1H), 3.35 (s, 3H), 2.35–0.79 (m), 0.98 (s, 3H), 0.70 (s, 3H); ^13^C NMR (100 MHz, CDCl_3_) δ 176.2, 140.7, 121.5, 94.6, 76.9, 59.6, 56.0, 55.1, 52.6, 50.1, 47.4, 41.9, 39.5, 38.8, 37.5, 37.2, 36.7, 32.2, 31.8, 31.7, 28.9, 27.8, 27.0, 25.0, 23.8, 22.7, 22.3, 20.8, 19.3, 14.2, 12.0.

#### *ent-*steroid 11

To a solution of *ent-*steroid 10 (236 mg, 0.5 mmol) in diethyl ether (20 ml), LiAlH_4_ (2.0 M in diethyl ether, 4.0 ml, 8.0 mmol) was added at room temperature. After 2 h, water (0.32 ml), 10% of NaOH (0.64 ml), and water (0.96 ml) were slowly added sequentially. After stirring for 30 min, the mixture was filtered through Celite and washed with CH_2_Cl_2_ (100 ml). The solvent was removed under reduced pressure and the residue was purified by flash column chromatography (silica gel eluted with 25% EtOAc in hexanes) to give *ent-*steroid 11 (212 mg, 98%): ^1^H NMR (400 MHz, CDCl_3_) δ 5.34–5.33 (m, 1H), 4.66 (s, 2H), 3.71–3.61 (m, 2H), 3.44–3.36 (m, 1H), 3.34 (s, 3H), 2.35–0.88 (m), 0.99 (s, 3H), 0.68 (s, 3H); ^13^C NMR (100 MHz, CDCl_3_) δ 140.6, 121.6, 94.6, 76.7, 62.5, 56.6, 55.1, 50.3, 50.1, 42.3, 42.0, 39.5, 39.1, 37.2, 36.6, 31.8, 29.5, 28.8, 27.9, 27.5, 24.1, 24.0, 22.7, 22.5, 21.0, 19.3, 12.1.

#### *ent-*steroid 12

To a solution of *ent-*steroid 11 (212 mg, 0.48 mmol) in CH_2_Cl_2_ (10 ml), mesyl chloride (1 mmol, 0.08 ml) and Et_3_N (0.28 ml, 2 mmol) were added at 0°C. After 1 h, aqueous NH_4_Cl was added and the product was extracted into CH_2_Cl_2_ (100 ml × 2). The combined extracts were dried over anhydrous Na_2_SO_4_ and filtered and the solvents removed. The residue was purified by flash column chromatography (silica gel eluted with 10% EtOAc in hexanes) to give *ent-*steroid 12 (241 mg, 97%): ^1^H NMR (400 MHz, CDCl_3_) δ 5.33–5.32 (m, 1H), 4.66 (s, 2H), 4.36–4.32 (m, 1H), 4.18–4.09 (m, 1H), 3.42–3.37 (m, 1H), 3.34 (s, 3H), 2.97 (s, 3H), 2.34–0.89 (m), 0.98 (s, 3H), 0.69 (s, 3H); ^13^C NMR (100 MHz, CDCl_3_) δ 140.6, 121.4, 94.6, 76.8, 70.0, 56.4, 55.1, 50.0, 49.9, 42.0, 39.7, 39.4, 39.2, 39.0, 37.2, 37.1, 36.6, 31.7, 31.6, 29.4, 28.8, 27.7, 27.4, 24.0, 23.4, 22.6, 22.4, 20.9, 19.3, 12.1.

#### *ent-*steroid 13

To a solution of *ent-*steroid 12 (241 mg, 0.46 mmol) in diethyl ether (30 ml), LiAlH_4_ (2.0 M in diethyl ether, 4.0 ml, 8.0 mmol) was added at room temperature. After 2 h, water (0.32 ml), 10% of NaOH (0.64 ml), and water (0.96 ml) were slowly added sequentially. After stirring for 30 min, the mixture was filtered through Celite and washed with CH_2_Cl_2_ (100 ml). The solvent was removed under reduced pressure and the residue was purified by flash column chromatography (silica gel eluted with 10% EtOAc in hexanes) to give *ent-*steroid 13 (188 mg, 95%): ^1^H NMR (400 MHz, CDCl_3_) δ 5.35–5.34 (m, 1H), 4.68 (s, 2H), 3.46–3.38 (m, 1H), 3.36 (s, 3H), 2.37–0.86 (m), 1.01 (s, 3H), 0.68 (s, 3H); ^13^C NMR (100 MHz, CDCl_3_) δ 140.7, 121.7, 94.6, 76.9, 56.7, 56.1, 55.1, 50.1, 42.3, 39.8, 39.5, 39.4, 37.2, 36.7, 36.2, 35.8, 31.9, 31.8, 28.9, 28.2, 28.0, 24.3, 23.8, 22.8, 22.5, 21.0, 19.3, 18.7, 11.8.

#### *ent-*steroid 14 (*ent-*cholesterol)

To a solution of *ent-*steroid 13 (188 mg, 0.44 mmol) in THF (20 ml), 6 N HCl (10 ml) was added at room temperature. After 4 h, the product was extracted into CH_2_Cl_2_ (100 ml × 2) and the combined extracts were washed with aqueous NaHCO_3_ (50 ml × 2), dried over anhydrous Na_2_SO_4_, and filtered. The solvent was removed under reduced pressure, and the residue was purified by flash column chromatography (silica gel eluted with 20% EtOAc in hexanes) to give *ent-*steroid 14 (165 mg, 98%); ^1^H NMR (400 MHz, CDCl_3_) δ 5.36–5.35 (m, 1H), 3.57–3.49 (m, 1H), 2.33–0.86 (m), 1.01 (s, 3H), 0.68 (s, 3H); ^13^C NMR (100 MHz, CDCl_3_) δ 140.7, 121.7, 71.8, 56.7, 56.1, 50.1, 42.3, 42.2, 39.8, 39.5, 37.2, 36.5, 36.2, 35.8, 31.9(2C), 31.6, 28.2, 28.0, 24.3, 23.8, 22.8, 22.6, 21.1, 19.4, 18.7, 11.8.

#### *ent-*steroid 15 (*ent-*4β-HC)

A procedure previously reported to convert cholesterol to 4β-HC was used (68) to convert *ent-*cholesterol 14 into *ent-*4β-HC 15.

To a solution of *ent*-cholesterol 14 (29 mg, 0.0747 mmol) in dioxane (5 ml) and water (2 drops), SeO_2_ (17 mg, 0.15 mmol) was added at room temperature. The mixture was heated to 90°C for 16 h. After cooling to room temperature, the solvent was removed under reduced pressure. The residue was purified by flash column chromatography (silica gel eluted with 30% EtOAc in hexanes) to give *ent*-4β-HC 15 (17 mg, 58%): mp 169–171°C; [α]_D_^20^ +41.7 (*c* = 0.12, CHCl_3_); ^1^H NMR (400 MHz, CDCl_3_) δ 5.69–5.68 (m, 1H), 4.15–4.14 (m, 1H), 3.58–3.55 (m, 1H), 2.20–0.78 (m), 1.19 (s, 3H), 0.69 (s, 3H); ^13^C NMR (100 MHz, CDCl_3_) δ 142.7, 128.8, 77.3, 72.5, 56.9, 56.1, 50.2, 42.3, 39.7, 39.5, 36.9, 36.2, 36.0, 35.8, 32.1, 31.8, 28.2, 28.0, 25.4, 24.2, 23.8, 22.8, 22.5, 21.0, 20.5, 18.7, 11.8; IR (film, cm^−1^) 3406, 1455, 1366, 1072.

## Supplemental data

This article contains supplemental data.

## Conflict of interest

R. Z. is cofounder, scientific advisor, and stockholder with Frontier Medicines Corp. All other authors declare that they have no conflicts of interest with the contents of this article.
