# Supplementary material for: 4β-Hydroxycholesterol is a prolipogenic factor that promotes SREBP1c expression and activity through the liver X receptor
Source: J Lipid Res. 2021 Feb 23;62:100051. doi: 10.1016/j.jlr.2021.100051 (PMC8042401; doi:10.1016/j.jlr.2021.100051)

Supplemental Figure 1:

A. 4β-HC and other LXR agonist do not affect AKT phosphorylation. Primary mice hepatocytes were treated o/n with vehicle 4β-HC, 24-HC or GW3965 followed with 40 minutes stimulation with Insulin. Proteins were extracted and AKT protein level was measured (N=1).

B. 4β-HC level do not change between feeding and fasting. Mice were fasted for 16hrs and then refed for 4 hours, followed by liver and serum collection. 4β-HC was measured by mass spectrometry (N=5).

C. 4β-HC does not induce ABCA1 in primary mice hepatocytes. Cells were treated o/n with vehicle or 5uM 4β-HC followed with 6hrs stimulation with Insulin. ABCA1 mRNA level was measured by RT-PCR (N=4).

Supplemental Figure 1

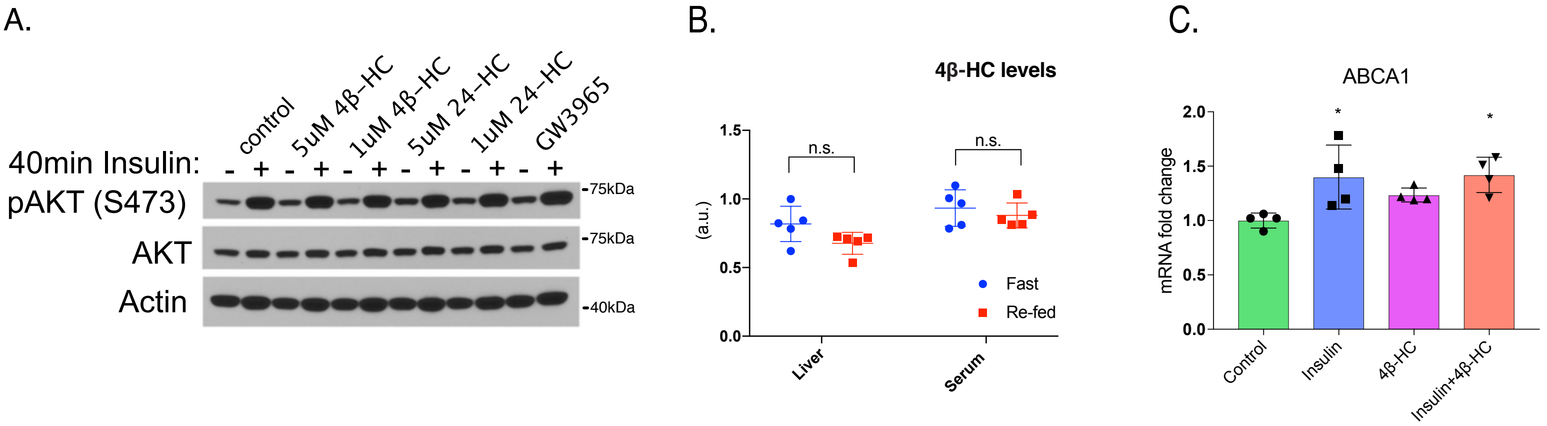

Supplement: Supplemental Fig. S1 [file mmc1.pdf]
